# Supplementary material for: Patterns of Intron Gain and Loss in Fungi
Source: PLoS Biol. 2004 Nov 30;2(12):e422. doi: 10.1371/journal.pbio.0020422 (PMC532390; doi:10.1371/journal.pbio.0020422)
Supplement: Table S1 — Also available at http://genes.mit.edu/NielsenEtAl/. (4.3 MB ZIP). [file pbio.0020422.st001.zip › NielsenEtAl/html/585.html]

AN5768.1.NCU03660.1.MG07482.1.FG07425.1


```
 CLUSTAL W (1.82) Multiple Sequence Alignments - Introns Inserted


Sequence 1: NCU03660.1	304 aa
Sequence 2: MG07482.1	307 aa
Sequence 3: FG07425.1	279 aa
Sequence 4: AN5768.1	275 aa
Alignment Length: 318 aa
Number Identitical Residues: 85 aa
Alignment Score (without introns) 4804


MG07482.1 	---MLRLSYW~VLPIISGVVWLA~TLLGLLLNWTVNEDRVHYGSMSENQTIA~YISDTGA
NCU03660.1	-----MPLFQ~IFPIISGVVWLG~TLLGLLIYWCTDENRRHYASMDARQTIA~YISDVGA
FG07425.1 	MAVKGVFSYW0WFPVLSGLVWLG~MLLGLLLEWQINQHGRRYPTQSIHSDVA~YISNVGA
AN5768.1  	---MWIISFW~IFPVISACMWIA1MLAAMLGTWCVQGYP-IYSSMEPGQTIA2YISDVGA
          	        :   :*::*. :*:.  * .:*  *  :     * : .  . :* ***:.**

MG07482.1 	YRLKPLFIAGCVITTIFLDAAFLADRWLRHKGRLVPNSTMGEKILSGL0TIAFAVVGTVG
NCU03660.1	STLKPLFIAGCSVTTVFLDLSFGADRWLRHKGRLVPNTTVTEKVLSGI~TIVFAIVGTCG
FG07425.1 	DRLWPLFITGCVLTSIFLDAAFLSERWLRHKGRLAPNTTLMEKVLTWL~SIAFAAVGTVG
AN5768.1  	QGLKPLFITGSVITVVFLDLSFVSERWLRHSGQLVPNKGRFDKFCAIA~SIFFSIAGALG
          	  * ****:*. :* :*** :* ::*****.*:*.**.   :*. :   :* *: .*: *

MG07482.1 	LIMLSIFDTASHNRLHNIFLSFFI~GGYTISAVFICWEYQRLG--~---R1NRDHRVLRI
NCU03660.1	LILLSIFDTARYPRLHDIFLLLFI~AGYVISAIFICWEYQRLGM-~---R~YREHRVLRI
FG07425.1 	LICLSIFRTGRYTRLHHTFLALFI~GGYLISAVFICWEYQRLGII1NPKD~YREHRVLRI
AN5768.1  	LILLSVYDTLRHSHLHSGFLAMFL2VAYLVSAILICIEYLRIGIF~---Y~RSQHRVLLA
          	** **:: *  : :**  ** :*:  .* :**::** ** *:*          :****  

MG07482.1 	SFWIKLVFIIVEVLLCVGFGIANRLR--EFNVAAVLEWI~IAVVFSFYVFSFYVDLYPAV
NCU03660.1	SFWVKLAFVVVEIILAVAFASCSYTG--HYNPAAVLEWT~IAFIFSFYIFSFWIDLYPAV
FG07425.1 	SFWVKLTFILVEVALIIAFGVTSRIK--QRNAAAILEWV~ISFIFTFYAISFVIDLYPAI
AN5768.1  	SFAIKAFFIVVEVALAIAFGICGWHRPHRRNAAAVLEWV1IALVFTFYVLSFVVDLLPSV
          	** :*  *::**: * :.*.  .   . . * **:***  *:.:*:** :** :** *::

MG07482.1 	YTKNGKPYTRPLAGGAFRTKG-MAEPPMAQRYRGGSASAAEEGRIDSDSTGAGGVGAAAA
NCU03660.1	RTKGTGGYRGNKGMNEHMEHGSREEQGIAGSDRTLMGEAYAAAHHGHDQRNSYYSGNGYS
FG07425.1 	RTKP-----------RHARHPKYVPSAIADS----SSPSHEGSRSDINDRRDVEMAQNTP
AN5768.1  	RTRN-----------HVPQGEKRINSAHSDHPEMVYEEPLTQDSAGPNANRNTNYYRGAH
          	 *:                  .      :         .      . :            

MG07482.1 	GGPFGSGAMSHTEREYYQRPPVPQNF
NCU03660.1	GSRPGTAPNQQSG---LRGMALPNDF
FG07425.1 	----------------------PRDF
AN5768.1  	V-------------------------
          	
```
